# Flight responses of eastern gray kangaroos to benign or harmful human behavior

**DOI:** 10.1002/ece3.5818

**Published:** 2019-11-26

**Authors:** Caitlin M. Austin, Daniel Ramp

**Affiliations:** ^1^ Centre for Compassionate Conservation Faculty of Science University of Technology Sydney Ultimo NSW Australia

**Keywords:** coexistence, countryside landscapes, eastern gray kangaroos, flight initiation distance, human behavior, human–wildlife interactions, hunting, *Macropus giganteus*, shooting

## Abstract

Globally, wilderness is being converted for rural and agricultural land use. In countryside landscapes, many habitat structures remain intact, providing suitable habitat for wildlife species that can accurately assess novel risks and develop tolerance to benign disturbances. Associative learning that promotes avoidance and also facilitates desensitization to benign disturbance is key to persisting in these landscapes. Conversely, learning to distinguish and avoid negative interactions with humans, like hunting, is vital. To determine if eastern gray kangaroos are capable of learning from previous interactions with humans, we tested the flight responses of wild kangaroos which have previously experienced either low or high frequencies of harmful and benign encounters with humans. We found that eastern gray kangaroos rapidly habituated to benign disturbance as there was no significant difference in assessment distance between groups that previously experienced low or high frequencies of disturbance. The threat of harmful disturbances was not as quickly learnt, as groups that experienced low frequencies of harmful disturbance delayed flight longer than those experiencing frequent harm. We found that the influence of environmental and group parameters on a kangaroo's decision to flee depended on the intent and frequency of previous interactions with humans. Our study indicates that kangaroos are learning from previous encounters with humans, correctly assessing novel risks which may be contributing to their persistence in countryside landscapes.

## Graphical abstract

Countryside landscapes provide novel resources and opportunities for species that can accurately assess the complexity of human activities, distinguishing between benign and harmful disturbance. This study found that eastern grey kangaroos are learning from previous interactions with humans; habituating to benign disturbances and perceiving humans as threatening when previous interactions have been harmful. Adaption to anthropogenic disturbances may be contributing to their persistence in these countryside landscapes.

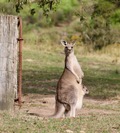

## 1 INTRODUCTION

Humans present a complex mix of negative and beneficial circumstances for many wildlife species. On one hand, the actions of humans have catastrophic unintended consequences for wildlife as their homes are either modified or occupied by development and land use (Fraser & MacRae, 2011). Wilderness, defined as areas that are mostly void of human presence, has declined by 9.6% in the last 20 years as the human population expands (Watson et al., 2016), while agriculture now utilizes roughly 30% of the ice‐free terrestrial land surface (FAO, 2012). However, where wildlife persist, they are increasingly challenged by having to accommodate humans in their daily routine (Soulsbury & White, 2016). For some, this creates novel opportunities to gain resources like exploiting waste and refuse (Gabrey, 1997; Ross, 2004), opportunities to share homes (Russell, Bowman, Herbert, & Kohen, 2011), and many positive interactions like supplemental feeding (Orams, 2002; Plummer, Risely, Toms, & Siriwardena, 2019). In contrast, many species find sharing space with humans makes life fraught and stressful (Ciuti et al., 2012). It stands to reason, that wildlife which adapt to, and persist within, anthropogenic landscapes, are able to balance the different benefits and costs associated with living with humans. Although there are a range of attributes and traits that promote the successful exploitation (or persistence) in these landscapes, the ability to accurately assess risk and respond accordingly is a key trait (Kretser, Sullivan, & Knuth, 2008; Lowry, Lill, & Wong, 2013; Samia, Nakagawa, Nomura, Rangel, & Blumstein, 2015).

Evidence of wildlife responding to human‐mediated fear in anthropogenic landscapes is strong. Some species avoid areas of high risk or else increase risk‐aversive behaviors to decrease risk propensity (Gaynor, Hojnowski, Carter, & Brashares, 2018; Rode, Farley, & Robbins, 2006; Tigas, Vuren, & Sauvajot, 2002), while others habituate to benign disturbances so that they may exploit favorable conditions (Sih, Ferrari, & Harris, 2011). Adaptation to urban environments by wildlife led to an appreciation of urban exploiters (Ducatez, Sayol, Sol, & Lefebvre, 2018; Fischer, Schneider, Ahlers, & Miller, 2015; Kark, Iwaniuk, Schalimtzek, & Banker, 2007; Soulsbury & White, 2016), wildlife who benefit from novel human‐dominated landscapes, requiring finely tuned behavioral strategies that permit risk avoidance but which do not inhibit cohabitation. Things are less clear in countryside environments, where there may be a mix of extant habitat and land cleared for agriculture (Daily, Ceballos, Pacheco, Suzán, & Sánchez‐Azofeifa, 2003). Human–wildlife conflicts are common in countryside environments, particularly when land sparing approaches to agriculture seek to exclude wildlife from productive land rather than integrating conservation with production (Dickman, 2010; Fischer et al., 2008). Methods of exclusion are often harmful to wildlife, which contributes to their perception of humans as threatening in these landscapes. Moreover, lower frequencies of human presence present challenging conditions for wildlife habituation while simultaneously disinhibiting negative human behavior (Thibaut, 2017). As a consequence, although the countryside can offer favorable conditions for wildlife, greater variation in human behavior excludes those species whose risk assessment is insufficiently sensitive and nuanced to accurately determine the risks humans pose in different circumstances.

For large mammals (>15 kg), evidence suggest many are resilient to minor modification of habitat in the countryside but commonly exhibit behavioral changes to avoid direct interactions with humans (Daily et al., 2003; Lawrence, 2008; Zhou et al., 2013). For these “avoiders,” encounters with humans are often perceived as threatening regardless of their intent or actions (Frid & Dill, 2002). However, the associative learning that promotes avoidance can also facilitate desensitization to benign disturbance (Stankowich, 2008), even though habituating to benign interactions must be complicated for species that also experience lethal human disturbances such as hunting. Despite this, there is growing evidence that some species can differentiate between contextual circumstances of harm and benign intent. Red deer (*Cervus elaphus*) have succeeded in making this distinction, perceiving recreationists as less threatening than hunters (Jayakody, Sibbald, Gordon, & Lambin, 2008). African elephants also exhibit stronger fear behaviors when presented with scent, visual, or audio stimuli from a threatening subgroup of people compared to that of an agricultural subgroup who poses little threat (Bates et al., 2007; McComb, Shannon, Sayialel, & Moss, 2014).

In Australia, eastern gray kangaroos (*Macropus giganteus*) are a large mammal faced with similar challenges: they are hunted by humans but also experience benign interactions with humans in recreational contexts. Eastern gray kangaroos are a gregarious woodland species (Caughley, 1964; Coulson, 2009; Kaufmann, 1975) that form open‐membership fission–fusion groups (Clarke, Jones, & Jarman, 1995; Jarman, 1987). Changes in group size have been attributed to perceived levels of predation risk which vary spatially and temporally (Heathcote, 1987; Jarman & Coulson, 1989). Eastern gray kangaroos increase group size when foraging in cleared landscapes during the morning and afternoon (Banks, 2001) then break into smaller groups during the middle of the day when the likelihood of predation decreases (Southwell, 1984). Eastern gray kangaroos are prey for foxes (primarily juveniles; Banks & Dickman, 2007) and dingoes (Letnic & Crowther, 2013; Wallach, Johnson, Ritchie, & O'Neill, 2010), but are also hunted by humans throughout their range. Indigenous people engaged in sporadic hunting of kangaroos for tens of thousands of years (Gammage, 2012). Since European occupation, kangaroos have been shot for food (for human and pets, commercially and for subsistence), sport, or bounties (Boom et al., 2012). The notion of hunting for sport is common as kangaroos in rural regions are often hunted illegally, a situation that is tolerated by government regulators (Boom & Ben‐Ami, 2013; Descovich, McDonald, Tribe, & Phillips, 2015; Ramp, 2013). However, interactions between humans and kangaroos are not always negative, as kangaroos can find safety and resources in national parks, golf courses, sporting ovals, and wildlife‐friendly farms (King, Wilson, Allen, Festa‐Bianchet, & Coulson, 2011). It appears that kangaroos are tolerant of, and habituate to, human disturbances of benign intent, such as tourism and wildlife‐friendly landholders (Austin & Ramp, 2019). It is unclear if these responses are caused by the frequency or intent of previous interactions with humans.

In a previous study (Austin & Ramp, 2019), we found that grouping behavior of eastern gray kangaroos varied in response to the intent and frequency of human disturbances. Under benign conditions, kangaroos formed larger groups when far from cover, following the “Many Eyes Hypothesis” (Ale & Brown, 2007; Beauchamp, 2013), but this relationship was not detectable under harmful conditions as group size did not change with distance to cover. This response was hypothesized as a behavioral adaption to human hunting as individuals learnt that forming large groups far from cover may make them targets for hunters (Austin & Ramp, 2019). Here, our goal was to test how these same kangaroos responded to the presence of a human stimulus to determine whether the intent and frequency of previous human–kangaroo interactions directly shaped kangaroo's fear of humans through associative learning. To test this, we conducted a flight response experiment on a population of free‐living kangaroos experiencing low and high frequencies of benign and harmful human disturbances (Austin & Ramp, 2019). If kangaroos learn from previous encounters with humans, we expected them to exhibit shorter assessment distances prior to flight when approached by a human when previous encounters were of harmful intent, relative to those who experienced encounters of benign intent. Incorrectly assessing risk of humans in countryside landscapes, like our study area, can have lethal consequences or result in lost foraging opportunities and increased energy expenditure. Additionally, we quantified the degree to which environmental and demographic parameters amplified risk perception by modeling the importance of distance to refuge, resource quality, group size, and demography on the group's decision to flee under each frequency and intent of human disturbance.

## 2 METHODS

### 2.1 Site description

**Figure 1:**
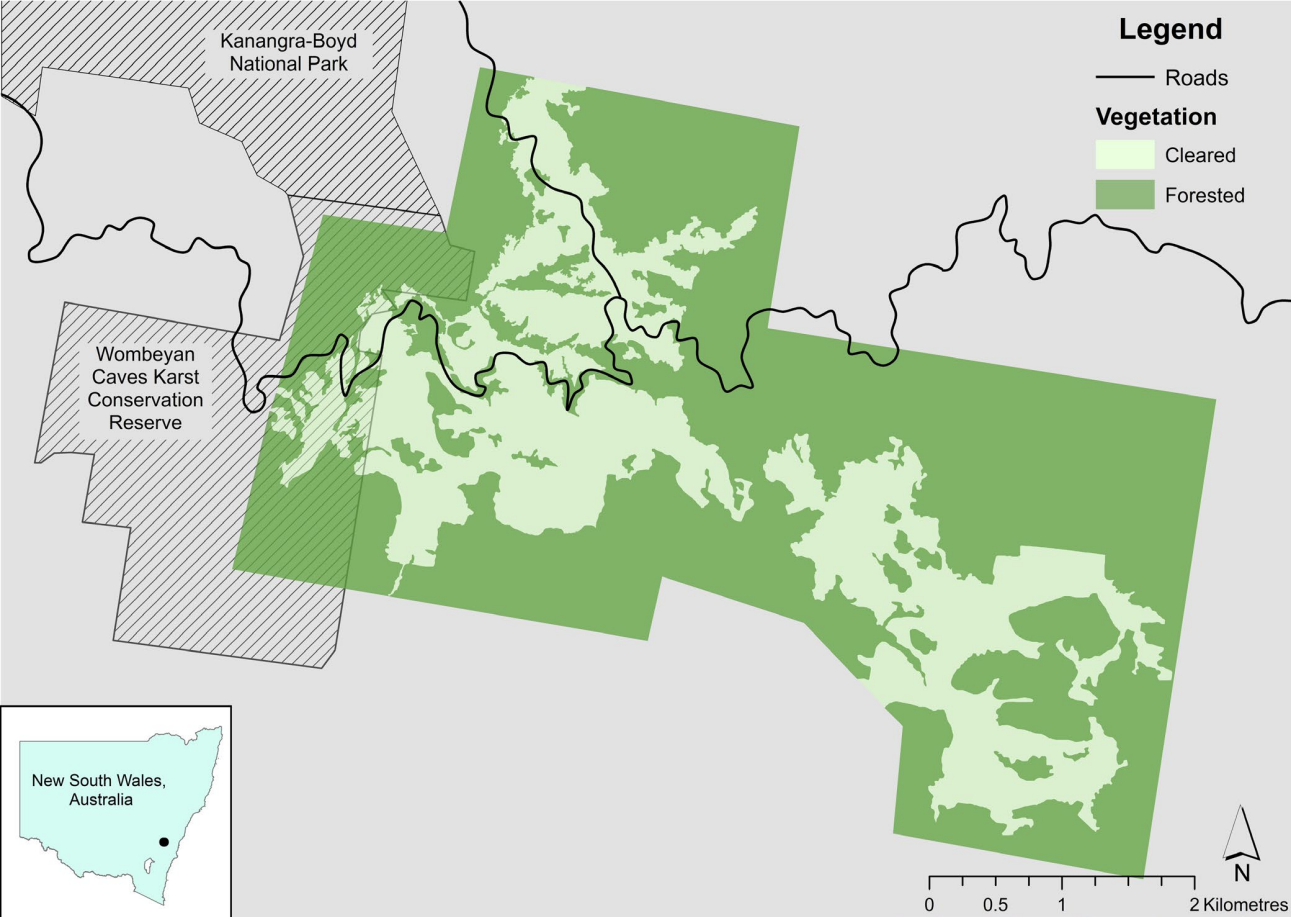
Location of study area within New South Wales, Australia, showing roads and forested and cleared areas within the study area. Property boundaries and human disturbance were omitted to ensure anonymity

We studied a free‐ranging population of eastern gray kangaroos in the surrounds of Wombeyan Karst Conservation Reserve in the Southern Highlands of NSW, adjacent to Kanangra‐Boyd National Park, previously described by Austin and Ramp (2019) (Figure 1). The area contains a mix of conservation reserve and private properties over 850 hectares, across which kangaroos are free to move. We previously established that the region included a mix of complex human presence, with areas of low (<1 kangaroo/human interaction per week) and high (>1 kangaroo/human interaction per week) frequency interaction, and a mix of benign (either ignored or well intentioned, e.g., tourists taking photographs) and harmful (harassing or shooting) interactions (Austin & Ramp, 2019). Consequently, we were able to classify regions by frequency and intent: High Benign (HB), Low Benign (LB), Low Harm (LH), and High Harm (HH). For the purposes of anonymity, we have not included map locations of each treatment. However, the study area was comprised of 4% HB, 47% LB, 21% LH, and 28% HH.

### 2.2 Behavioral responses

Measuring direct responses to fear can be inferred from observation of antipredator behaviors such as flight, vigilance, grouping, and crypsis, helping to identify and quantify stimuli that trigger fear responses. Flight response to a stimulus is frequently relied upon in wildlife studies and can be recorded in a variety of ways (Miller, Garner, & Mench, 2006). Flight initiation distance (FID), the distance at which an animal flees from an approaching stimulus, is highly correlated with alert distance (AD), the distance at which prey become aware of the stimulus, and the distance from which the stimulus approach commenced (Dall, Houston, & McNamara, 2004; Dumont, Pasquaretta, Réale, Bogliani, & Hardenberg, 2012). Alert behaviors can be difficult to identify in some species as there may be no clear indicators of stimulus detection. However, alert postures in kangaroos are clearly observable as they become upright, standing high on their hind legs, and focus their attention (eye and ear orientation) in the direction of the disturbance (Edwards, Best, Blomberg, & Goldizen, 2013). Alert distance allows for the more insightful assessment distance (Dall et al., 2004) to be measured, the distance a stimulus can move toward an animal after it has been detected until flight is taken. This measure directly relates to perceived predation risk as it reflects the period where threat level changes from low to high risk (Frid & Dill, 2002; Stankowich & Blumstein, 2005; Ydenberg & Dill, 1986). This measure has previously been used to quantify perceived risk by Columbian black‐tailed deer (*Odocoileus hemionus columbianus*) to different types of threats (speed of approach, directness, and presence of gun; Stankowich & Coss, 2005).

We therefore determined flight responses of 138 groups of eastern gray kangaroos by measuring assessment distance across the four types of human disturbance. We sampled flight responses from each disturbance type over six fortnight windows, between October 2016 and February 2017, recording responses between 06:00–08:30 and 16:30–19:00 when kangaroos were grazing in open areas (Clarke et al., 1995). We covertly located groups of more than one individual, selecting groups for testing to ensure the same individuals were not recorded twice in the same sampling session (although individuals were not identifiable between sessions). Before testing flight responses, video of the group was recorded using a digital camera (Canon EOS 70D Digital SLR with Canon EF 100–400 mm lens) for 3 min to ensure they had not detected our presence. The GPS coordinates (±5 m) of the starting location were recorded along with the distance between the starting location and the most central individual in the group, using a laser rangefinder (Bushnell, ±0.9 m). The test commenced as the human stimulus (CMA) walked in a direct line toward the group, keeping the group in sight but avoiding eye contact. The approacher maintained a constant speed during the approach (0.7 ± 0.03 m/s) and always wore the same clothing. Following Stankowich and Coss (2005), a marker was dropped when one or more members of the group displayed a vertical vigilance stance toward the approacher (alert distance). The approacher continued without stopping until one or more individuals moved from their original position (flight initiation distance), concluding the test. We recorded the final location of the approacher and the dropped marker using a GPS. The exact position of the group was determined using the directional bearing, start location, and initial distance of the group.

### 2.3 Environmental and group parameters

Eastern gray kangaroos use forested habitat as a refuge and forage closer to cover when predation risk is high (Banks, 2001). The group's distance from forested cover was calculated from the GPS position at the center of the group in ArcGIS (v10; ESRI, 2016). We measured the resource quality at the center of each group by determining the relative green channel brightness (greenness) of vegetation from digital photographs. Due to the high correlation between greenness and biomass (Inoue, Nagai, Kobayashi, & Koizumi, 2015), resource quality was inferred by the mean greenness of resources for each group of kangaroos as per Austin and Ramp (2019). Using video footage collected prior to the approach, we assigned individuals to demographic categories; size/maturity (pouch young, young‐at‐foot, sub‐adult, small adult, medium adult, and large adult (Austin & Ramp, 2019). The presence of all pouch young was noted but were only recorded as contributing to group size when they were out of their mother's pouch. An independent assessor familiar with eastern gray kangaroos was provided with a subset of 100 photographs to help validate our categorization of demographic groups. Group size was determined using the 15‐m chain rule, where individuals within 15 m of another member of the group were included in the group (Best, Dwyer, Seddon, & Goldizen, 2014; Carter, Pays, & Goldizen, 2009; Jarman, 1987; Pays, Beauchamp, Carter, & Goldizen, 2013).

### 2.4 Statistical analysis

We examined AsD using a generalized linear mixed model to detect significant differences between our four disturbance types (High Benign, Low Benign, Low Harm, and High Harm), with inference determined using likelihood ratio tests. Sampling session was included as a random variable to control for possible dependence due to repeated sampling of sites. However, parametric bootstrapping found sampling session had no significant effect on AsD. Multiple comparisons of means with the Tukey contrasts were conducted to test for statistical differences between disturbances. To determine if the presence of vulnerable individuals (mothers, pouch young, and young‐at‐foot) significantly affected AsD we ran a series of independent sample *t* tests within each disturbance type. The response variable AsD was log transformed to satisfy assumption of homogeneity of variance. Linear regressions were conducted to determine if the distance at which kangaroos were alerted to the approach (AD) had a significant effect on AsD, nested within disturbance type. We conducted an analysis of covariance to identify significant interactions between disturbance type and AsD, controlling for AD. This analysis was repeated with each disturbance type set as the reference level.

The effect of environmental and group parameters on AsD was tested using multiple model inferencing. The global model for AsD included the following predictors: proportion of individuals from each demographic category, group size, distance to refuge, and resource greenness. All variables were standardized and scaled to remove bias (Grueber, Nakagawa, Laws, & Jamieson, 2011). For each disturbance type, a set of models were generated from all combinations of predictors using the R package “MuMIn” (Barton & Barton, 2018). Models for each treatment were ranked according to AICc and all models within 2 + AICc of the best model were averaged using the natural average method (Burnham & Anderson, 2002). Coefficients and confidence intervals were generated from full averaged models.

## 3 RESULTS

**Figure 2:**
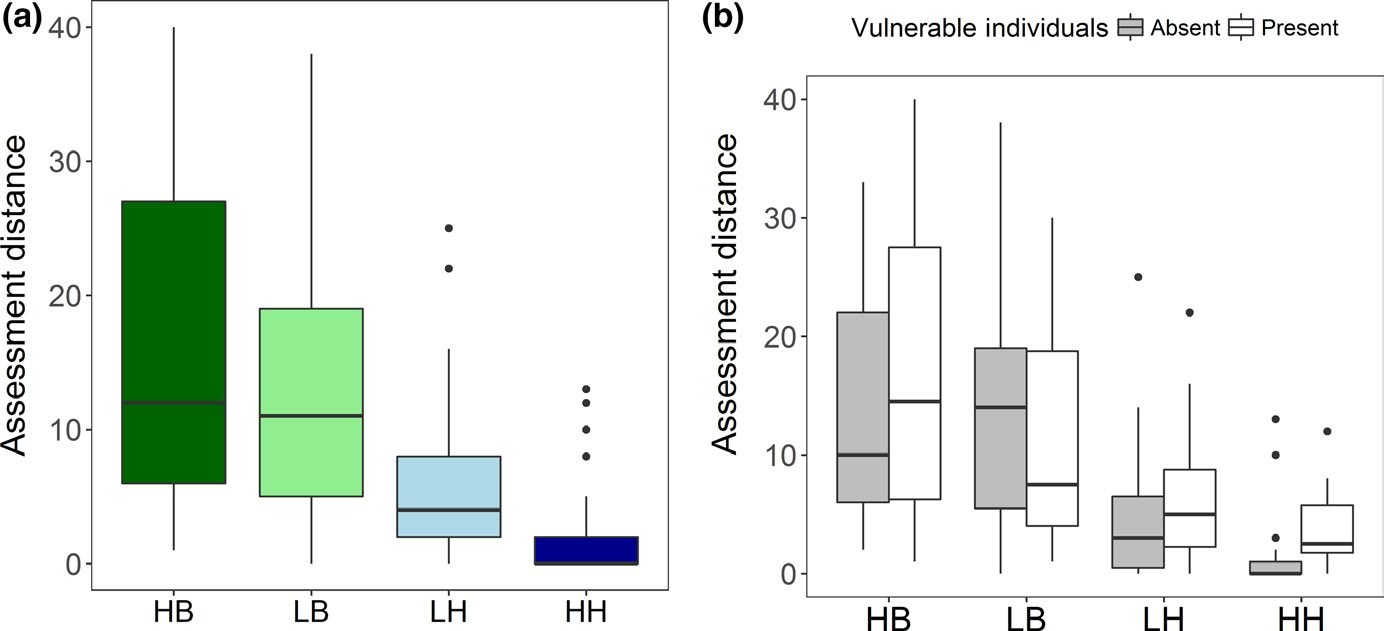
(a) Mean assessment distances for groups of eastern gray kangaroos under different human disturbances, HB: High Benign, LB: Low Benign, LH: Low Harm, and HH: High Harm. (b) Mean assessment distances for groups of eastern gray kangaroos as a function of human disturbance and the presence of vulnerable individuals (pouch young and young‐at‐foot). Width of boxes is proportional to the square root of the sample sizes. Shaded boxes represent groups without vulnerable individual and hollow boxes groups containing vulnerable individuals

Human disturbance type significantly influenced the assessment distance of eastern gray kangaroos (*p* < .001). The frequency of benign human interactions had no significant effect on assessment distance (HB: LB, *p* = .638), with average assessment distances of 16.17 m (±2.02) and 12.73 m (±1.55), respectively. However, assessment distances were significantly longer at both LB and HB than for groups at LH (*p* = .001, *p* < .001) and HH (*p* < .001, *p* < .001; Figure 2a). The frequency of harmful interactions with humans significantly affected assessment distance, with mean assessment distances at HH of 2.21 m (±0.70), which were significantly shorter than those at LH by 3.76 m (±1.09) on average (*p* = .004; Figure 2a). The proportion of groups with vulnerable individuals present (young‐at‐foot, and pouch young) varied across disturbance types; vulnerable individuals were present in 94% of the groups sampled at HB; 41% at LB; 33% at LH, and 29% at HH. At HH, the presence of vulnerable individuals in a group resulted in a mean assessment distances that were 2.8 times longer than when vulnerable individuals were absent (4.13–1.48 m, *t*_27_ = −2.671, *p* = .013). However, the presence of vulnerable individuals had no significant effect on assessment distance at all other treatments (HB: *t*_33_ = −0.671, *p* = .507; LB: *t*_39_ = 0.947, *p* = .3494; LH: *t*_31_ = −0.942, *p* = .353; Figure 2b).

**Figure 3:**
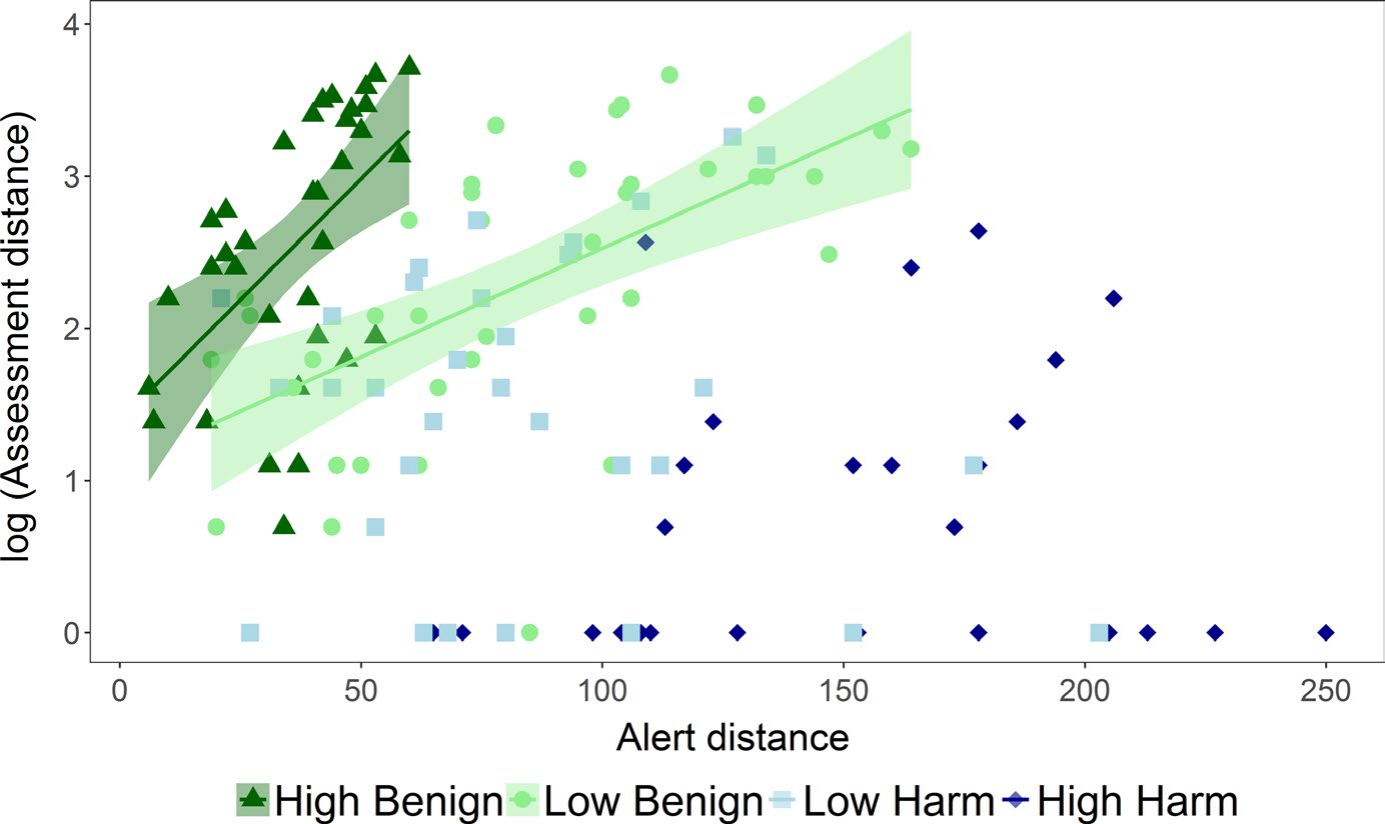
Relationship between logged assessment distance and alert distance under different human disturbances, HB: High Benign, LB: Low Benign, LH: Low Harm, and HH: High Harm. Linear trend lines were plotted for significant relationships with shaded regions reflecting confidence intervals (95%)

Alert distance was positively correlated with assessment distance for kangaroos that have previously experienced benign disturbances (HB: *f* = 13.48, *p* = .001; LB: *f* = 24.33, *p* < .001), such that kangaroos could afford to spend longer assessing threat levels when the detected threat was further away (Figure 3). This relationship was similar for both benign disturbance types, as the slopes for HB and LB were not significantly different (*f* = 17.81, *p* = .1). In contrast, no significant linear relationship between alert distance and assessment distance for groups that experience harmful disturbances was detected (LH: *f* = 0.26, *p* = .611; HH: *f* = 0.11, *p* = .741), suggesting that the decision to flee at harmful sites was independent of how far away the threat was (Figure 3).

**Figure 4:**
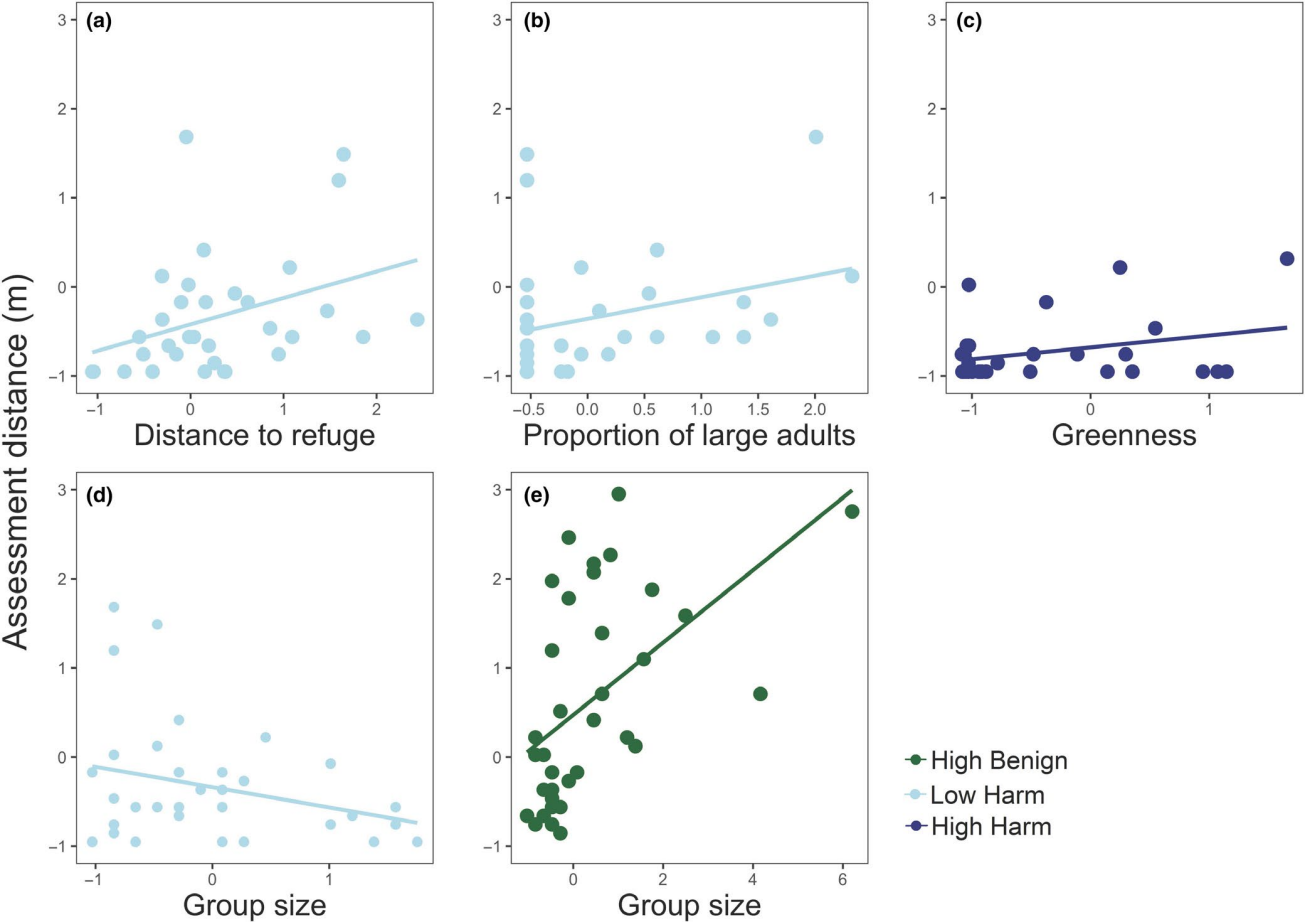
Significant responses of assessment distance to environmental and group parameters across disturbance types. Variables have been scaled to allow comparison across variables. Relationship between assessment distance and (a) distance to refuge at LH, (b) the proportion of large adults in the group at LH, (c) resource greenness at HH, (d) group size at LH, and (e) group size at HB

There was considerable difference in the influence of environmental and grouping variables across disturbance types (Table 1; Figure 4a). Distance to refuge was an important positive predictor of assessment distance at LH (*β* = .380, *p* = .003), with those further from refuge taking longer to assess threat. There was also a weak trend at LH where the presence of large adults in the group also increased the length of assessment distance (*β* = .315, *p* = .016; Figure 4b). However, increasing group size led to shorter assessment distances at LH (*β* = −.288, *p* = .022; Figure 4d). Conversely, increasing group size led to significantly longer assessment distances at HB sites (*β* = .349, *p* = .001; Figure 4e). At HH sites, kangaroos took longer to assess threats (i.e., were more reluctant to leave) when plant quality (i.e., resource greenness) was higher (*β* = .179, *p* = .015; Figure 4c).

**Table 1:** Average model summaries of assessment distance across different human disturbances, High Benign, Low Benign, Low Harm, and High Harm

Disturbance	Parameter	Estimate^a^	Adjusted *SE*	*p* value	Relative importance
High Benign *n* = 35	Intercept	0.638	0.381	.102	NA
	Large adult	–	–	–	–
	Medium adult	–	–	–	–
	Small adult	0.065	0.141	.652	0.29
	Sub‐adult	−0.025	0.090	.784	0.14
	Young‐at‐foot	0.018	0.088	.844	0.10
	Pouch young	–	–	–	–
	**Group size**	**0.394**	**0.118**	**.001**	**1**
	Distance to refuge	0.200	0.387	.612	0.32
	Resource greenness	–	–	–	–
Low Benign *n* = 41	Intercept	0.199	0.147	.191	NA
	Large adult	−0.020	0.075	.790	0.9
	Medium adult	0.319	0.179	.082	0.91
	Small adult	0.024	0.089	.793	0.16
	Sub‐adult	−0.060	0.143	.680	0.26
	Young‐at‐foot	−0.095	0.122	.443	0.52
	Pouch young	0.012	0.064	.858	0.8
	Group size	–	–	–	–
	Distance to refuge	0.016	0.070	.823	0.14
	Resource greenness	–	–	–	–
Low Harm *n* = 33	Intercept	−0.494	0.108	<.001	NA
	**Large adult**	**0.315**	**0.121**	**.016**	**1**
	Medium adult	–	–	–	–
	Small adult	–	–	–	–
	Sub‐adult	–	–	–	–
	Young‐at‐foot	0.023	0.064	.729	0.26
	Pouch young	–	–	–	–
	**Group size**	**−0.288**	**0.121**	**.022**	**1**
	**Distance to refuge**	**0.380**	**0.123**	**.003**	**1**
	Resource greenness	–	–	–	–
High Harm *n* = 29	Intercept	−0.474	0.115	<.001	NA
	Large adult	−0.126	0.107	.244	0.68
	Medium adult	−0.183	0.143	.205	0.68
	Small adult	0.052	0.084	.545	0.32
	Sub‐adult	0.034	0.102	.742	0.14
	Young‐at‐foot	−0.116	0.275	.680	0.21
	Pouch young	0.064	0.103	.539	0.32
	Group size	0.173	0.118	.153	0.84
	Distance to refuge	–	–	–	–
	**Resource greenness**	**0.179**	**0.115**	**.015**	**1**

Note

Statistically significant variables at 95% confidence level are shown in bold. A dash indicates that the variable was not present in the model.

a Effect sizes are standardized.

## 4 DISCUSSION

We found that the nature and frequency of previous interactions with humans shaped risk perception in eastern gray kangaroos. Empirical results suggest that kangaroos whose primary experiences with humans are benign encounters, at both high and low frequencies, perceive an experimental human stimulus as less threatening than those who have experienced harmful interaction with humans. The frequency of benign interactions did not significantly alter assessment distance, which implies that tolerance of benign human disturbances is readily learnt, even when the disturbance is encountered infrequently. However, the frequency of past harmful experiences with humans significantly affected their perception of risk. Kangaroos that experienced disturbance at low frequencies spent longer assessing the potential threat than those who experienced higher frequencies of harmful disturbance, which flee almost immediately after the human stimulus was detected. When previous interaction is benign, our results align with the general notion that birds, mammals, and lizards learn that a nonthreatening stimulus poses little to no threat after several encounters with the stimulus (Delacasa & Lubow, 1995; Gonzalo, López, & Martín, 2013; Samia et al., 2015). In our study, low levels of benign disturbance also resulted in tolerance. Previous research has shown that repeated presentation of a consistently benign stimulus leads to rapid habituation, for example in marmosets (Dacier, Maia, Agustinho, & Barros, 2006) and bears (Elfström, Zedrosser, Støen, & Swenson, 2014). Habituating to benign disturbance has economic benefits, enabling individuals to avoid the costs of fleeing nonthreatening disturbances; namely the loss of resources and unnecessary expenditure of energy (Ydenberg & Dill, 1986).

Flight behavior and risk assessment in eastern gray kangaroos in response to people has received little academic focus. Previous studies of macropod flight behavior have used flight initiation distance to detect changes in antipredator behavior following the loss of predators on islands (Blumstein, 2002; Blumstein & Daniel, 2005) or to investigate the role of flight behavior in vehicle collisions (Lee, Croft, & Ramp, 2010). Our study found that distance to refuge, resource quality, group size, and group demography all variously influenced assessment distance across disturbance types. Generally, prey are more fearful when safety is further away (Bonenfant & Kramer, 1996; Dill & Houtman, 1989). However, we found that kangaroos spent longer assessing the threat before fleeing when they were further from safety. We propose that this is likely due to the energy costs of fleeing further to reach safety. Monitoring the disturbance stimulus for longer allows kangaroos to make an accurate assessment of the potential risk before incurring energetic costs.

Group size is known to have a highly variable effect on assessment distance across species (Stankowich & Blumstein, 2005), and, as our study showed, this effect can also be influenced by the nature of previous interactions with humans. For example, larger groups at Low Harm sites exhibited shorter assessment distances than those with fewer individuals, while the opposite was found at High Benign sites where there was a positive correlation between assessment distance and group size. The trend at sites with harmful interactions may be explained by the notion that some individuals in a group will have had negative experiences with humans, making them less inclined to delay fleeing one a threat has been noticed. On the other hand, at benign sites, increasing assessment distance with group size fits well with the notion that individuals perceive lower levels of risk when in a larger group, as the likelihood of a given individual falling prey to a predator is reduced when more individuals are present (Carter et al., 2009; Jarman, 1987). This effect has been observed in similar‐sized herbivore species such as deer (De Boer, Breukelen, Hootsmans, & Wieren, 2004) and caribou (Aastrup, 2000).

The demographic composition of groups also influenced flight response. Large adult kangaroos typically spent longer assessing the stimulus, as adults may be choosing to dedicate more time to assessing the threat in order to negate the energetic cost of fleeing, which is higher for larger animals (Norberg, 2012). Likewise, groups containing vulnerable young also spent significantly longer assessing risk than those composed only of adults at sites of high harm, but not at benign sites. This finding is contrary to our initial expectations, where we expected that groups with vulnerable individuals would respond quicker to risk in threatening landscapes (Blumstein, 2010; Cooper & Blumstein, 2013; Stankowich, 2008). The delay in flight could be due to the higher energetic needs of young and mothers (Cripps, Wilson, Elgar, & Coulson, 2011; Gélin, Wilson, Coulson, & Festa‐Bianchet, 2013), as these groups might not wish to abandon foraging opportunities until the threat is confirmed to be imminent (Cooper et al., 2003; Stankowich & Blumstein, 2005; Ydenberg & Dill, 1986). This explanation is supported by our finding that resource quality also influenced assessment distance at High Harm sites. When foraging in areas with high quality resources, eastern gray kangaroos reduce the amount of time spent on antipredator behaviors such as vigilance (Favreau, Goldizen, Fritz, & Pays, 2018). Similar reductions of antipredator behavior have also been observed for impalas, which were less vigilant when patch quality was high (Pays et al., 2012). A second possibility is that in this threatening landscape, flight itself might increase risk, particularly for vulnerable individuals and their guardians.

Studies of flight responses of ungulates have found that many species spend more time assessing threats if they were alerted to the disturbance further away (Stankowich & Coss, 2005). This gives prey the opportunity to process additional information about the risk to more accurately assess the level of threat posed, enabling appropriate antipredator behaviors to be selected (Cárdenas, Shen, Zung, & Blumstein, 2005). Our findings supported this explanation under benign conditions, like at camp grounds, as kangaroos habituate to human presence, leading to groups expressing smaller spatial zones of risk. In these circumstances, kangaroos learn that monitoring potential threats and delaying flight incurs little increased risk. In contrast, this response broke down when past disturbances were harmful. Disturbances like shooting remain a risk from a greater distance, which could explain the lack of correlation between assessment distance and alert distance in landscapes where previous interactions with humans have involved shooting. The adaption of wildlife to human hunting has been widely reported, where wildlife exhibit stronger fear responses toward humans in threatening scenarios, for example, during hunting season (De Boer et al., 2004; Jayakody et al., 2008; Matson, Goldizen, & Putland, 2005). Hunting has also had a marked effect on wildlife behavior, which sees animals modifying activity patterns and their use of habitats (Bonnot et al., 2013; Lone, Loe, Meisingset, Stamnes, & Mysterud, 2015; Manor & Saltz, 2003; Saïd, Tolon, Brandt, & Baubet, 2012). Our findings suggest that kangaroos have learnt more than just when and where humans pose a significant threat but have also developed responses to mitigate these novel risks. Similar modification of antipredator behavior was observed by Austin and Ramp (2019), where kangaroos modified their antipredator grouping in response to human hunting. Behavioral changes in hunted populations may be attributed to the selection of individuals which possess beneficial traits that facilitate survival (Ciuti et al., 2012; Sol, Lapiedra, & González‐Lagoset, 2013). In order for the trends we detected to be attributed to selection, the entire population would have to experience widespread and sustained hunting in order to eliminate individuals with unfavorable characteristic. It is unlikely that human disturbances at our study site were sufficiently intensive to alter behaviors through selection.

Our study indicates that kangaroos are learning from their previous interactions with humans, rapidly habituating to benign human disturbances and identifying humans as a threat when previous interactions were harmful. The ability to modify antipredator behaviors and correctly assess risk of humans in countryside landscapes can provide foraging opportunities and habitat in a time where wilderness is decreasing at an astonishing rate. Our exploration of how environmental and group parameters affected kangaroo's fear of humans will inform future studies in understanding the ways in which kangaroos are persisting in countryside habitats when faced with novel threats and opportunities.

## CONFLICT OF INTEREST

The authors declare no conflict of interest.

## AUTHOR CONTRIBUTIONS

Conceptualization (C.M.A. and D.R.), investigation (C.M.A.), formal analysis (C.M.A. and D.R.), visualization (C.M.A. & D.R.), writing—original draft (C.M.A.), writing—review and editing (D.R.), supervision (D.R.), funding acquisition (D.R.).

## Data Availability

Data supporting this study are available from the Dryad Digital Repository https://doi.org/10.5061/dryad.mcvdncjwb
